# PEG-fibrinogen hydrogel microspheres as a scaffold for therapeutic delivery of immune cells

**DOI:** 10.3389/fbioe.2022.905557

**Published:** 2022-08-09

**Authors:** Noam Cohen, Yaron Vagima, Odelia Mouhadeb, Einat Toister, Hila Gutman, Shlomi Lazar, Avital Jayson, Arbel Artzy-Schnirman, Josué Sznitman, Arie Ordentlich, Shmuel Yitzhaki, Dror Seliktar, Emanuelle Mamroud, Eyal Epstein

**Affiliations:** ^1^ Israel Institute for Biological Research, Ness-Ziona, Israel; ^2^ Department of Biomedical Engineering, Technion-Israel Institute of Technology, Haifa, Israel

**Keywords:** PEG-fibrinogen, hydrogels, cell delivery, *Yersinia pestis*, airways-on-chip

## Abstract

Recent advances in the field of cell therapy have proposed new solutions for tissue repair and regeneration using various cell delivery approaches. Here we studied *ex vivo* a novel topical delivery system of encapsulated cells in hybrid polyethylene glycol-fibrinogen (PEG-Fb) hydrogel microspheres to respiratory tract models. We investigated basic parameters of cell encapsulation, delivery and release in conditions of inflamed and damaged lungs of bacterial-infected mice. The establishment of each step in the study was essential for the proof of concept. We demonstrated co-encapsulation of alveolar macrophages and epithelial cells that were highly viable and equally distributed inside the microspheres. We found that encapsulated macrophages exposed to bacterial endotoxin lipopolysaccharide preserved high viability and secreted moderate levels of TNFα, whereas non-encapsulated cells exhibited a burst TNFα secretion and reduced viability. LPS-exposed encapsulated macrophages exhibited elongated morphology and out-migration capability from microspheres. Microsphere degradation and cell release in inflamed lung environment was studied *ex vivo* by the incubation of encapsulated macrophages with lung extracts derived from intranasally infected mice with *Yersinia pestis*, demonstrating the potential in cell targeting and release in inflamed lungs. Finally, we demonstrated microsphere delivery to a multi-component airways-on-chip platform that mimic human nasal, bronchial and alveolar airways in serially connected compartments. This study demonstrates the feasibility in using hydrogel microspheres as an effective method for topical cell delivery to the lungs in the context of pulmonary damage and the need for tissue repair.

## Introduction

Cell-based therapies are promising advanced type of medical treatments that allow the repair and regeneration of damaged tissues and organs. Yet, most current delivery strategies of cell transplants in the clinics have mainly used direct injections of cells in buffer, providing insufficient protection for the cells. As a result, the vast majority of transplanted cells lose viability during their delivery, leading to relatively low functional engraftment yields (Li et al., 2009; Sheikh et al., 2012). To overcome these limitations, different bioengineering approaches have emerged using injectable hydrogels based on various biomaterial components (Wang et al., 2019). Specifically, hybrid biosynthetic hydrogels having both naturally occurring components and synthetic components provide structural support and biological activity (Birman and Seliktar, 2021). This artificial niche promotes cell survival by protecting the cells from microenvironment assaults, such as high shear stress and inflammation (Mitrousis et al., 2018), as well as by preventing cell loss at the time of delivery. Moreover, hydrogels have been shown to actively support cell processes such as morphogenesis, cell migration, cell differentiation and cell adhesion (Almany and Seliktar, 2005).

The hybrid natural/synthetic hydrogel Polyethylene glycol (PEG)-fibrinogen (PEG-Fb) has previously been used in different biotechnological (Cohen et al., 2018) and therapeutic tissue engineering applications (Peled et al., 2007; Fuoco et al., 2012b; Rufaihah et al., 2017). Local injection of encapsulated cells in PEG-Fb has enhanced cell survival (Fuoco et al., 2012a) and migration into surrounding host tissues (Seeto et al., 2017). However, distal delivery of encapsulated cells in PEG-Fb microspheres, such as systemic administration intra venously or topically to respiratory tracts, has not been demonstrated yet.

In this study, we investigated *in vitro* the potential in topical delivery of encapsulated cells within PEG-Fb hydrogel microspheres to respiratory tract in inflamed and damaged environment, such as following bacterial infection. To that end we used a pneumonic plague model of mice intranasally infected with *Yersinia pestis* bacterium to obtain inflamed whole lung extracts and assessed their effects on cell viability, cytokine expression and mobilization from the hydrogel microspheres *ex vivo*. As a proof of concept for topical delivery of encapsulated cells to human respiratory tracts, we utilized a recently developed multi-component Airways-on-chip models that mimic human nasal, bronchial and alveolar airways (Nof et al., 2022).

## Materials and methods

### Cell lines

Murine derived MH-S and J774 cell lines were obtained from ATCC. TC-1 is a tumor cell line derived from primary lung epithelial cells of C57BL/6 mice (Lin et al., 1996). This cell line was a kind gift from the laboratory of Prof. T.C. Wu (Johns Hopkins University). Cells were cultured in full media containing DMEM (Biological Industries) supplemented with 10% (v/v) Fetal Calf Serum (FCS) (Biological Industries), 0.2% (v/v) penicillin/streptomycin (Pen/Strep) (Biological Industries, Israel) and 2 mM L-glutamine (Biological Industries, Israel). Trypan blue reagent (Biological Industries, Israel) was used to count cells and to determine cell viability.

### Fabrication of PEG-Fb hydrogel microspheres

PEG-Fb precursor molecules (8 mg/ml protein and 108% PEGylation) were synthesized as described previously (Almany and Seliktar, 2005; Dikovsky et al., 2006). Microspheres were prepared using a photo-initiator emulsion-based method as was described previously (Cohen et al., 2018) with some modifications. PEG-Fb precursor solution was prepared by mixing PEG-Fb molecules with 1% (v/v) of 10% (w/v) pluronic F68 (Merck, Israel) in PBS, 1.5% (v/v) triethanolamine (TEOA), 0.39% (v/v) of N-vinyl pyrrolidone (NVP) (Merck, Israel) and 1% (v/v) of 10 mM eosin Y photo-initiator (Merck, Israel) in PBS. PEG-Fb precursor solution was mixed with 2 × 10^6^ cells in a total volume of 100 µl and transferred to 1 ml mineral oil (Merck, Israel) in a glass test tube. The tube was vigorously vortexed for 2 s to form an emulsion and immediately subjected to photo-crosslinking using white light lamp (150 W) for 40 s. The emulsion was washed twice with 2 ml of full media (centrifuge at 200 g for 5 min). The oil phase was discarded, and the pellet microspheres were resuspended in full media, seeded on 6-well plates and incubated at 37°C and 5% CO_2_ for 30 min to allow non-encapsulated cells to attach to the plate. Supernatants containing the microspheres were taken for further use. For delivery assay purposes, microspheres were filtered using pluriStrainer with 150 µm cutoff (PluriSelect, 43-50150-03), and the filtrate was further used.

### Live/dead assay of encapsulated cells

To examine the viability of the encapsulated cells, microspheres were washed with PBS (1,200 rpm, 5 min) and resuspended in 1 ml PBS containing 1 µl Calcein AM (Merck, Israel) and 5 µl ethidium bromide solution (Merck, Israel). After 15 min incubation at 37°C, the microspheres were washed with PBS and visualized in Nikon Ts2 fluorescence inverted microscope or in LSM 710 confocal scanning microscope (Zeiss, Jena, Germany) equipped with the following lasers: argon multiline (458/488/514 nm), diode 405nm, DPSS 561 nm and helium-neon 633 nm. To determine the number of live encapsulated cells, ten *z*-axis images (range of 200 µm) were acquired from three different random positions and analyzed in ImageJ. The number of live and dead cells were counted within each microsphere and the viability percentiles were determined.

### Examination of cytokine secretion from lipopolysaccharide-exposed macrophages

J774 macrophages were cultured in 24-well plates (500,000 cells/well) and incubated overnight in 37°C. The next day, *E. coli* LPS (Merck, Israel) was added in a final concentration of 10 ng/ml. Cell counts and viability determination were performed every day for 4 days using trypan blue. Supernatants were taken each day to determine secretion of TNFα in an enzyme-linked immunosorbent assay (ELISA) according to the manufacturer’s protocol (R&D Systems, MN, United States).

### Animal procedures

All animal experiments were performed in accordance with the recommendations for the Care and Use of Laboratory Animal (National Institute of Health [NIH]) and Israeli law and were approved by the Israel Institute for Biological Research Animal Care and Use Committee (Permit Number: IACUC-IIBR M-28-2013). Female C57BL/6 mice (6–10 weeks old) were purchased from Invigo Israel (Rehovot, Israel). Mice were infected intranasally (i.n) with *Y. pestis* virulent strain Kimberley53 (Kim53) as described previously (Vagima et al., 2015). Briefly, bacterial colonies were harvested and diluted in heart infusion broth (HIB) (BD, United States) supplemented with 0.2% xylose and 2.5 mM CaCl_2_ (Merck, Israel) to an OD_660_ of 0.01 and grown for 22 h at 28°C in a shaker (100 rpm). The bacterial culture was diluted in PBS to the required infectious dose based on pre-determination of bacterial concentration by counting colony forming units (CFU) after being plated on BHIA plates (48 h at 28°C). Prior to infection, mice were anesthetized with a mixture of 0.5% ketamine HCl and 0.1% xylazine and then infected i.n. with 35 μl/mouse of the bacterial suspension, whereas naïve mice were administered i.n. with PBS only.

Whole lung mRNA extractions were obtained as previously described (Vagima et al., 2019). Briefly, mice were euthanized, and lungs were then removed and placed on a 70-μm nylon cell strainer (BD Falcon, United States) dipped in 2 ml PBS containing 1% protease inhibitor cocktail (Merck, Israel). The mashed tissue was centrifuged (1,200 rpm, 5 min), the pellet of cells in suspension was used for total RNA extraction and the supernatant was filtered in 0.2 µm filter and used for microsphere degradation experiments.

### Microsphere degradation

Hydrogel microspheres loaded with microphages were cultures in 24-well plates (50,000 cells/well) in 1 ml culture medium supplemented with 300 µl whole lung extract (not diluted) of *Y. pestis* infected mice or control mice. Hydrogel degradation was monitored for 7 days, and the number of degraded microspheres (determined as clustered cells without boundaries) was counted in quadruplets. The degradation percentage was determined using three independent experiments.

### Determination of mRNA expression in extracted tissues

Lung cell suspensions were prepared as described above. Total RNA was extracted from whole lung cell suspensions using Tri-reagent (Merck, Israel) according to the manufacturer’s instructions. Two micrograms of total RNA were reverse-transcribed using Moloney murine leukemia virus reverse transcriptase and oligo-dT primers (Promega, United States). Quantitative PCR analysis was performed using an ABI 7500 instrument (Applied Biosystems, United States) with SYBR green PCR master mix (Applied Biosystems, United States). The fold change in the quantity of gene transcripts was measured and compared to the hypoxanthine phosphoribosyl transferase (HPRT) gene using the comparative (−2^ΔΔCt^) method. Forty cycles of PCR amplification were performed in duplicate for each primer set. Primer sequences used are listed in Table 1.

**TABLE 1:** Primer sequences.

Mouse gene	Forward 5′-3′	Reverse 5′-3′
MMP3	CTT​CCC​AGG​TTC​GCC​AAA​AT	CAT​GTT​CTC​CAA​CTG​CAA​AGG​A
MMP7	GGT​GAG​GAC​GCA​GGA​GTG​AA	GCG​TGT​TCC​TCT​TTC​CAT​ATA​ACT​TC
MMP8	CAC​ACA​CAG​CTT​GCC​AAT​GC	TCC​CAG​TCT​CTG​CTA​AGC​TGA​AG
MMP9	CAGACGTGGGTCGATTCC	TCATCGATCATGTCTCGC
MMP14	CCCAAAAACCCCGCCTAT	TCT​GTG​TCC​ATC​CAC​TGG​TAA​AA
TNFα	CAT​CTT​CTC​AAA​ATT​CGA​GTG​ACA​A	TGG​GAG​TAG​ACA​AGG​TAC​AAC​CC
IL1β	CAA​CCA​ACA​AGT​GAT​ATT​CTC​CAT​G	GAT​CCA​CAC​TCT​CCA​GCT​GCA
HPRT-1	AGTACAGCCCCAAAATGG	TCC​TTT​TCA​CCA​GCA​AGC​T

### H&E staining and confocal microscopy

For hematoxylin and eosin (H&E) general histopathology evaluation, lungs were rapidly isolated, and fixed in 4% neutral buffered PFA at room temperature for 1 week, followed by routine processing for paraffin embedding. Serial sections, 5 µm thick, were performed and selected sections were stained with H&E for light microscopy examination. Images were acquired using 3D HISTECH panoramic midiII3.0.0. For confocal microscopy analysis, images were acquired using a Zeiss LSM710 confocal microscope (Zeiss, Oberkochen, Germany).

### Delivery of encapsulated cells to respiratory tract models using airways-on-chip technology

To illustrate hydrogel delivery into respiratory airway tracts, we used a recently established a multi-compartment *Airway-on-chip* model that recapitulates key anatomical and physiological components of the human respiratory tracts (Nof et al., 2022). These include three distinct compartments of nasal passages, bronchial and acinar (alveoli) airways, mimicking the extra-thoracic, conductive and respiratory regions, respectively (Artzy-Schnirman et al., 2019; Elias-Kirma et al., 2020; Sznitman, 2021). Briefly, 3D-printed molds were used to fabricate the nasal and bronchial airway compartments, and standard soft-lithography techniques combined with a modified method for master production using dry reactive ion etching (DRIE) of silicon on an insulator wafer were used to manufacture the small (<100 µm) features characteristic of the acinar model. The resulting molds were used as a master template for polydimethylsiloxane (PDMS) casting. PDMS was prepared according to the manufacturer’s instructions and poured into the appropriate templates. Cured PDMS was subsequently peeled from the molds and punched using a biopsy punch of 1 mm size to create inlet and outlets. Each of the three airway compartments were connected via silicon tubing. A peristaltic pump was connected to the inlet of the nasal device allowing perfusion of a media containing hydrogel microspheres (200 hydrogels/ml) at a physiologically relevant flowrate (10 ml/min) for 20 min. A Y-joint tube-to-tube connector was used in the outlet of the bronchial device to reduce the flowrate fed to the acinar model, based on the physiologically relevant range mimicking airflows at such airway depths.

## Results

### Optimization of cell encapsulation in PEG-Fb hydrogel microspheres

**FIGURE 1:**
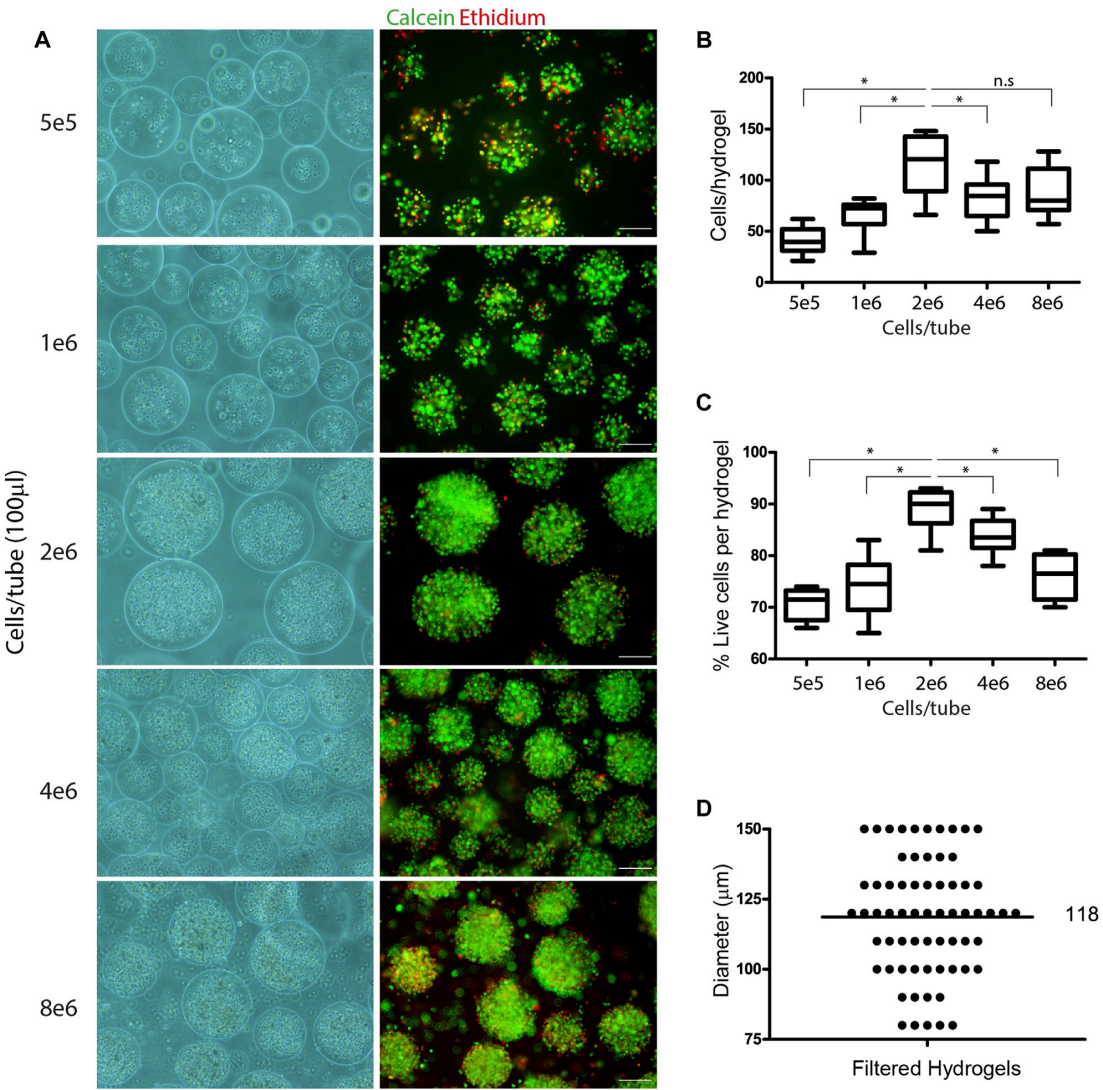
Co-encapsulation of alveolar macrophages (MH-S) and epithelial (TC1) cells inside hydrogel microspheres. **(A)** Representative brightfield and fluorescent images of encapsulated MH-S cells in elevated number of cells per reaction (5 × 10^5^-8x10^6^ cells/100 µl in a glass tube). Encapsulated cells were stained with Calcein (green) and ethidium (red) for live/dead assay. Scale bar 100 µm. **(B)** Box plot showing median of encapsulated cell number per hydrogel microspheres using elevated number of cells per reaction tube (5 × 10^5^-8x10^6^ cells/tube). The presented results are from ten individual microspheres, a representative from three independent experiments. **(C)** Box plot showing live cell percentiles inside microspheres measured by live/dead assay in elevated number of cells per reaction tube (5 × 10^5^-8x10^6^ cells/tube). The presented results are from ten individual microspheres, representative of three independent experiments. **(D)** Dot plot showing microsphere diameter after filtration using 150 µm cutoff filter. The average of 118 µm is shown in black line. The presented results are from three different experiments in which each experiment was represented by at least twenty individual microspheres that were measured. *p value < 0.05 tested in Wilcoxon non-parametric test; n.s, not significant.

The design principle of injectable microsphere delivery systems to the respiratory airways is based on the ability to target the delivery of encapsulated cells to the site of interest, while preserving their viability and function. This includes the delivery of cells with different functional properties, such as macrophages that can mediate immune responses or stem cells that can promote the replenishment and recovery of damaged tissues. In some specific therapeutic procedures, it would be beneficial to deliver to the site of damaged tissue two or more cell types that could induce synergic biological effects. In such scenarios, the encapsulation could be taking place either for one or two (and possibly more) cell types together inside a microsphere carrier. Co-encapsulation of different cell types would be particularly advantageous when close interactions are required for intercellular activities. Here we examined the encapsulation of MH-S cells, an alveolar macrophage cell line, alone and together with TC1 cells, an alveolar epithelial cell line. Microspheres with encapsulated cells were prepared using a photo-initiator emulsion-based method that involves the mixture of cells in a tube together with PEG-Fb precursors (see *methods*). The concentration of cells inside the reaction tube determines the final number of encapsulated cells per microsphere. To examine the optimal number of macrophages needed to achieve cell-packed microspheres, different amounts of cells per glass tube (5 × 10^5^ to 8 × 10^6^ cells per 100 µl in the glass tube) were photo-crosslinked by an emulsification polymerization reaction. The microspheres appeared to be packed with cells when 2 × 10^6^ cells/reaction (or more) were used (Figure 1A). The encapsulation process resulted in increased numbers of cells per microsphere from 40 cells, using 5 × 10^5^ cells/reaction, to 125 cells, using 2 × 10^6^ cells/reaction. Then the number of cells per microsphere remained similar at 4 × 10^6^ and 8 × 10^6^ cells/reaction (Figure 1B). The viability of encapsulated cells was tested with a live/dead assay using Calcein, a fluorescent dye of live cells (green), and Ethidium, a fluorescent dye of dead cells (red). We found that the use of 2 × 10^6^ cells per reaction was optimal and resulted in 90% viability compared to lower or higher cell amounts (Figure 1C). The encapsulation process resulted in a wide diameter range of microspheres from 50 to 300 µm diameter. To adjust the microsphere diameter to further experimental delivery into respiratory tract *in vitro* models, we used a cell strainer to filter out large microspheres. Using a 150 µm filter provided us with microspheres in the range of 75–150 µm with average of 118 µm in diameter (Figure 1D).

**FIGURE 2:**
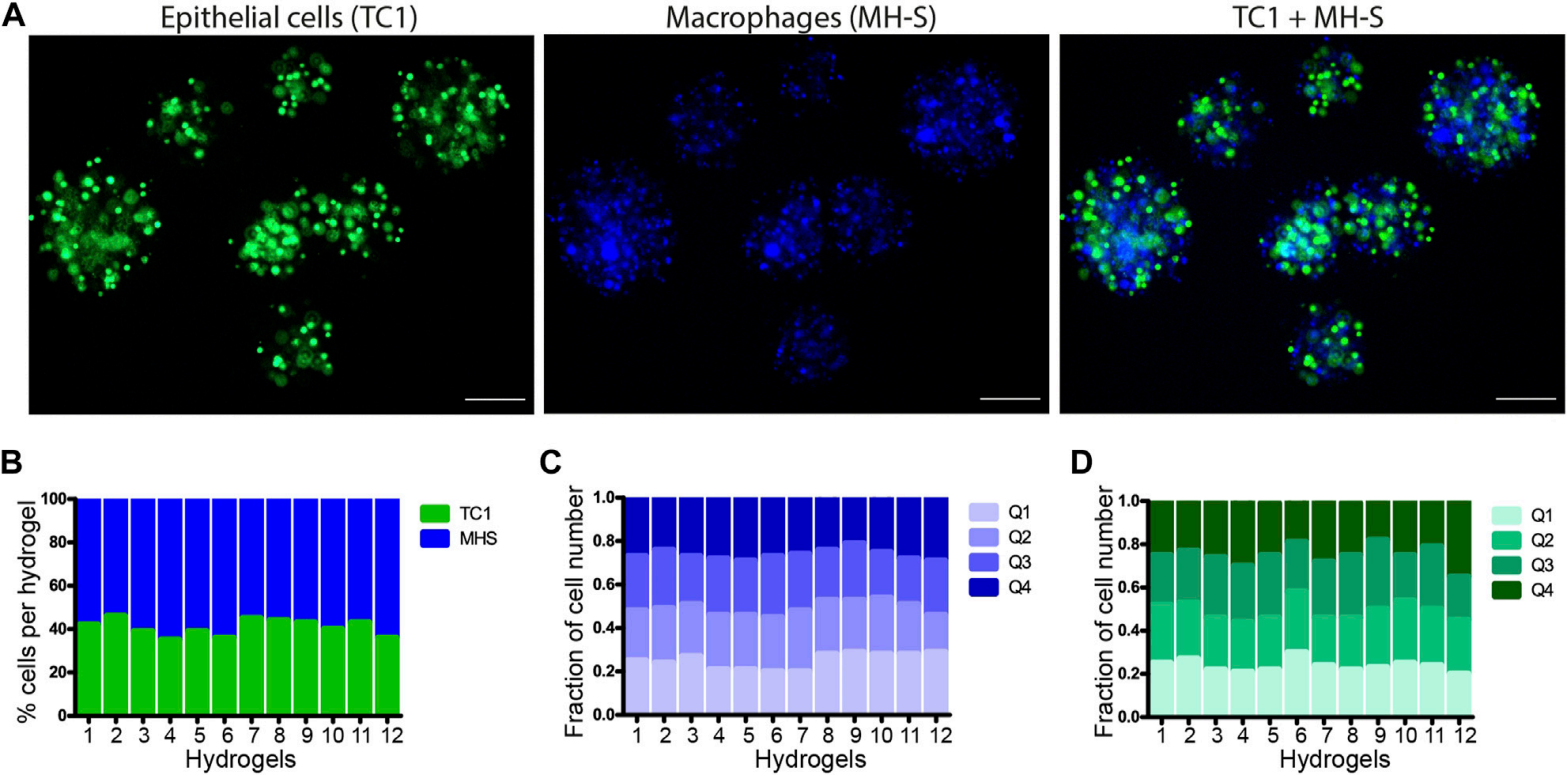
Equal distribution of two co-encapsulated cells inside the microspheres. **(A)** Representative fluorescent images of alveolar epithelial TC1 cells and macrophage MH-S cells encapsulated alone or together in 1:1 proportion (TC1 + MH-S). **(B)** Bar plot showing percentiles of TC1 (green) and MH-S (blue) cells inside twelve different microspheres. **(C,D)** Bar plot showing the fraction of MH-S cells **(C)** and TC1 cells **(D)** per quarter (Q1-Q4) inside twelve different microspheres.

To examine the encapsulation of two types of cells in the same microsphere, we used two different cellular dyes. TC1 cells were stained with Calcein, a fluorescent dye staining cell cytoplasm, and MH-S cells were stained with DAPI, a fluorescent dye staining the nuclei. Then an equal portion of both cells (1:1) was taken for encapsulation. Figure 2A shows fluorescent images of the co-encapsulation of TC1 and MH-S cells in the microspheres. To examine the number of TC1 or MH-S cells inside the microsphere, we counted the cells in each image channel separately and merged the results. The mean proportions of TC1 and MH-S cells inside the microspheres were 40.6 ± 3.7% and 60.5 ± 3.7%, respectively (Figure 2B), demonstrating relatively equivalent proportions of both cell types. To measure the distribution of the cells inside the microsphere, we used the microscope software to divide microsphere images into four quarters (Q1-Q4) and counted the cells in each quarter. The fraction of cells in each quarter out of total ranged between 0.23 ± 0.02 to 0.25 ± 0.04 (Figures 2C,D), demonstrating equal distribution of the co-encapsulated cell types inside the microspheres.

### Encapsulated macrophages are protected from endotoxin exposure and migrate out of the microspheres upon activation

**FIGURE 3:**
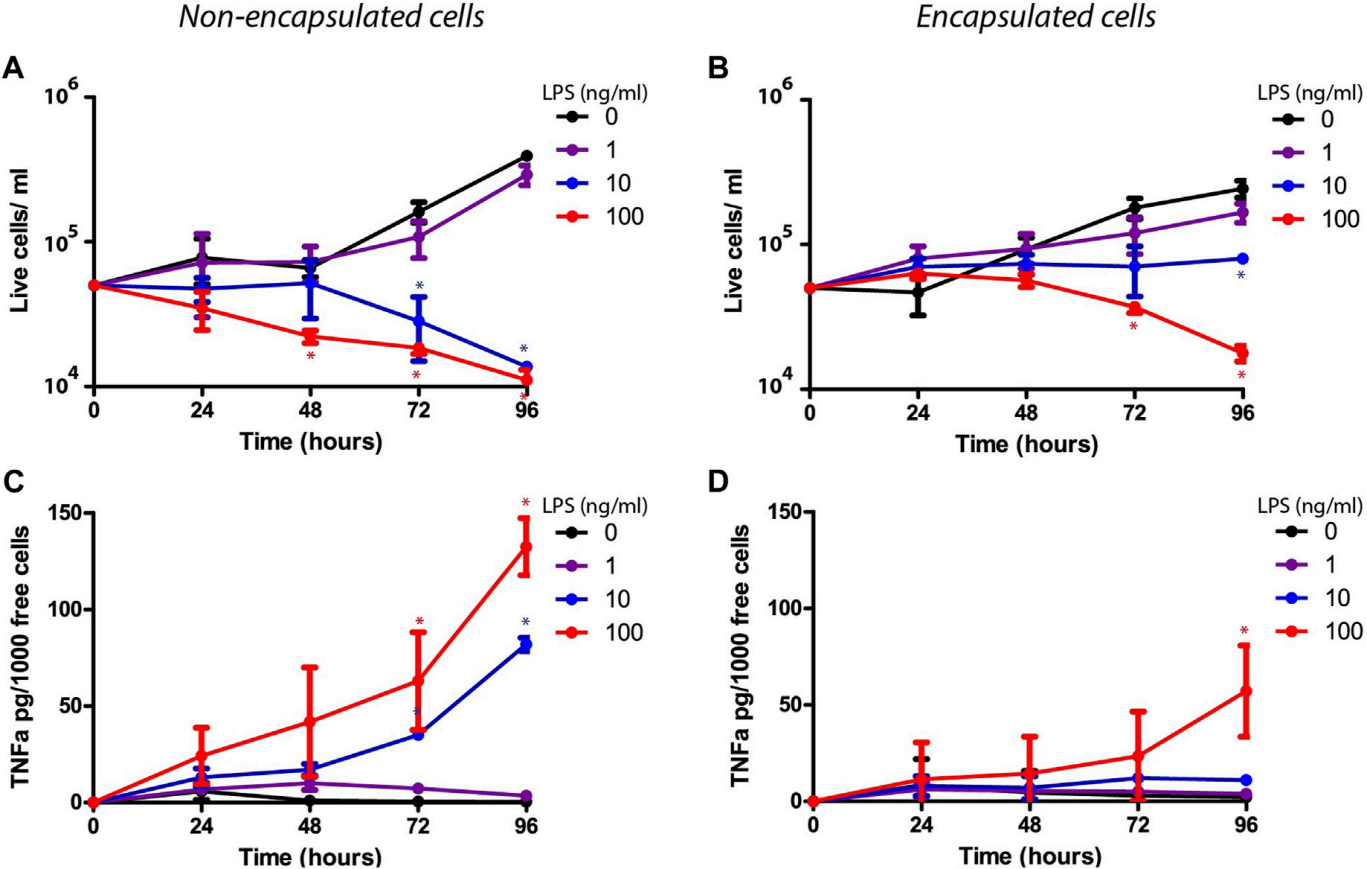
The effect of LPS on TNFα secretion by encapsulated vs. non-encapsulated macrophages. **(A,B)** Concentrations of free (non-encapsulated) **(A)** and encapsulated macrophages **(B)** in 24-well plates over time after exposure to 1, 10, 100 ng/ml LPS (purple, blue and red line) or none (black line). **(C,D)** Amounts of TNFα secretion per 1,000 free macrophages and encapsulated macrophages after LPS stimulation after exposure to LPS. *p value < 0.05 tested in Wilcoxon non-parametric test.

**FIGURE 4:**
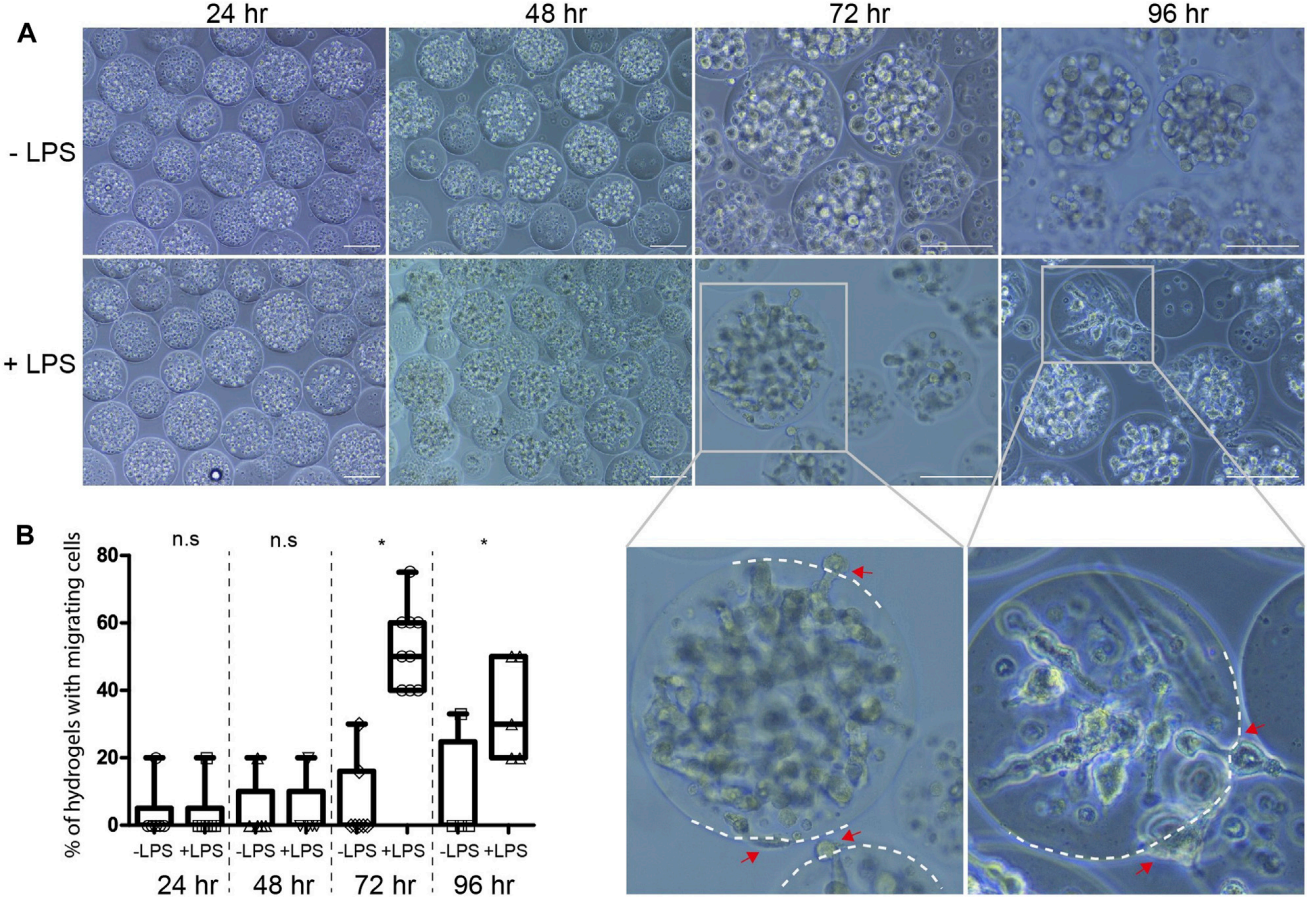
The effect of LPS on cell migration outside the microspheres **(A)** Brightfield images of encapsulated macrophages that were LPS-exposed or not exposed over the course of 96 h. Scale bar 100 µm. Magnified images show cell migration outside the microsphere margins (marked in dashed white lines). **(B)** Box plot showing percentiles of hydrogels with migrating cells calculated from six different images for each LPS or non-LPS exposed cells. *p value < 0.05 tested in Wilcoxon non-parametric test; n.s, not significant.

Macrophages are specialized immune cells that mediate phagocytosis and cytokine secretion as part of the host response to invading pathogens (Hussell and Bell, 2014). Their pivotal role in maneuvering the immune response may provide an advantageous therapeutic approach to treat lung diseases in early stages of the disease (Suzuki et al., 2014). We argued that the PEG-Fibrinogen microspheres would give a supportive and protective environment to the encapsulated macrophages from the bacterial endotoxic environment, and accordingly would preserve their viability and function. To test that, we examined *in vitro* the effect of exposure to *E. coli* lipopolysaccharide (LPS) on viability and cytokine secretion of encapsulated macrophages compared to free non-encapsulated macrophages. To that end, J774 type macrophages were chosen, due to their strong response to bacterial LPS. Encapsulated and free macrophages were cultured with a dose response of 1, 10 and 100 ng/ml *E. coli* LPS for 96 h and analyzed each day for cell viability and secretion of TNFα, a major pro-inflammatory cytokine secreted from macrophages upon the exposure to LPS. Both encapsulated and free non-encapsulated macrophages had similar viability, cell growth and secreted low baseline levels of TNFα under none or minor LPS exposure (1 ng/ml), whereas both exhibited reduced cell viability and increased TNFα secretion under high concentrations of LPS (100 ng/ml) (Figures 3A–D). In contrast, the incubation with an intermediate level of LPS (10 ng/ml) revealed different responses; encapsulated cells were significantly highly viable and secreted only minor amounts of TNFα, whereas non-encapsulated cells were less viable and had a burst TNFα secretion (Figures 3A–D). These results suggest that the effect of encapsulation of macrophages protected against the bacterial endotoxic environment in a dose dependent manner, prevented the typical burst cytokine response, and retained a relatively low-activate state of the cells. An observation on encapsulated cell morphology revealed that LPS-exposed encapsulated cells (10 ng/ml) became elongated and migrated outside the hydrogel microsphere (Figure 4A, red arrows). This migration was two-fold higher when compared to non-LPS exposed encapsulated cells (Figure 4B), suggesting that under LPS activation, the encapsulated macrophages are capable to migrate out of the microspheres.

### *Ex vivo* induction of microsphere degradation using lung extracts of *Y. pestis* infected mice

To examine the concept to deliver encapsulated macrophage to inflamed environment of infected lungs with bacteria, we used lung extracts from a previously established mouse model of pneumonic plague (Levy et al., 2010; Vagima et al., 2015) and test them on encapsulated macrophages *ex vivo*. Intranasal infection with highly pathogenic bacterium *Y. pestis* induces an inflammatory cytokine storm in the lungs of infected mice, bacterial dissemination to internal organs and death without an early antibiotic treatment (Tomashefski Jr, 2000). Moreover, respiratory infection with *Y. pestis* induces severe tissue damages associated with the secretion of proteolytic enzymes such as matrix-metalloproteases (Vagima et al., 2015, 2020). Under such inflammatory conditions, the delivery and release at site of infection of encapsulated macrophages could be valuable to mediate the resolution of inflammation.

**FIGURE 5:**
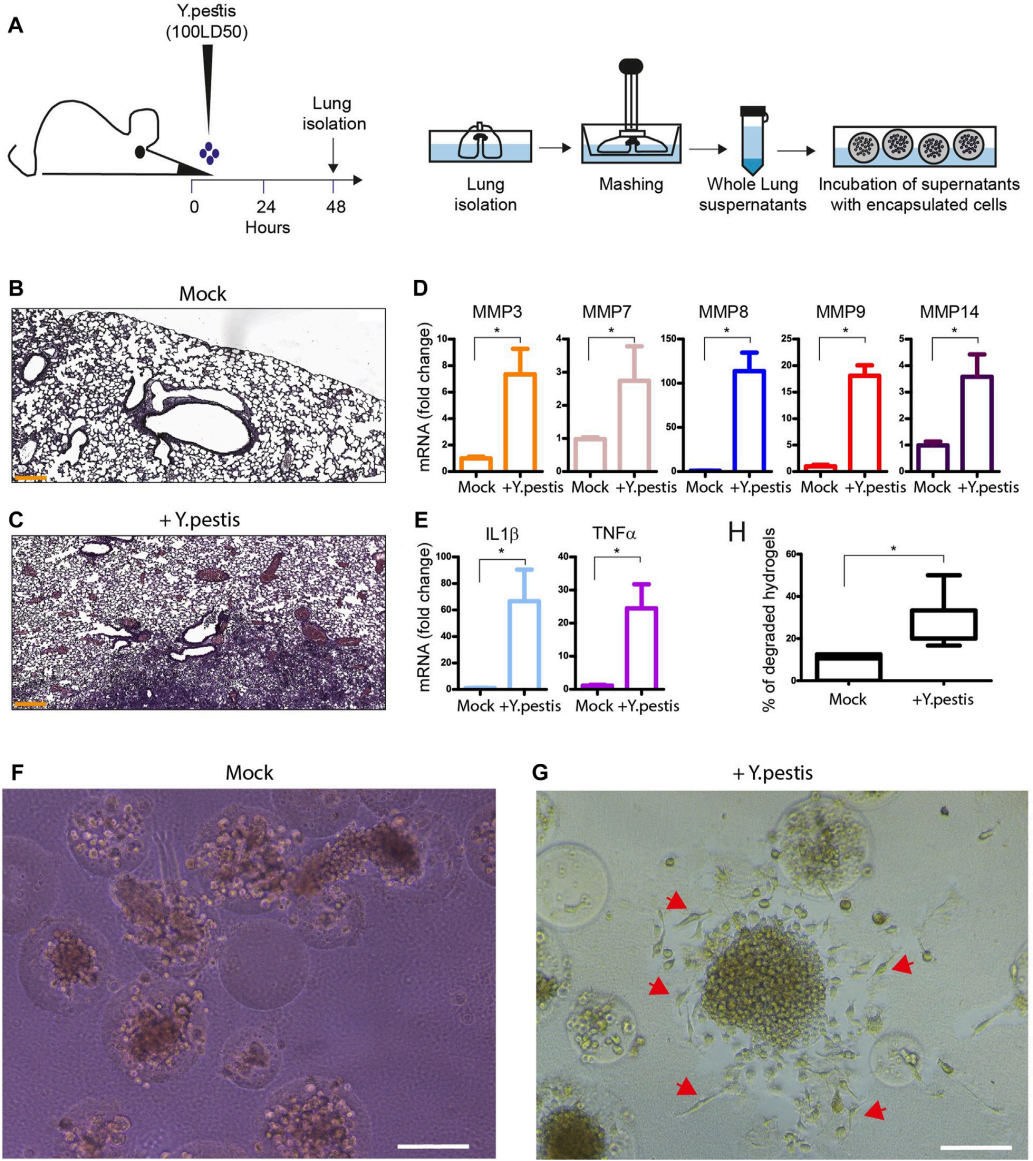
*Ex vivo* incubation of microspheres with Y. pestis infected whole lung extracts. **(A)** An illustration of the experimental design showing intranasal administration of a dose of 100LD_50_ (110,000 CFU) of *Y. pestis* bacteria (Kim53 strain). After 48 h, the mice were sacrificed, and the lungs were isolated. Isolated lungs were mashed and the whole lung supernatants were filtered and transferred to cultures of encapsulated macrophages in microspheres. **(B,C)** Hematoxylin and eosin staining of lung sections from representative mock **(B)** and *Y. pestis*-infected mice **(C)**, 48 h post infection. **(D,E)** Bar plots showing mRNA expression (fold change) of metalloproteases MMP3, 7, 8, 9 and 14, as well as, IL1β and TNFα cytokines in mock or *Y. pestis*-infected (+Kim53) lungs, 48 h post infection. **(F,G)** Brightfield images of encapsulated macrophages in microspheres after 7 days incubation with mock extract **(F)** or *Y. pestis*-infected lung extracts **(G)**. **(H)** Box plot showing the percent of degraded hydrogels in incubation with mock- and *Y. pestis*-infected lung extracts. Results are presented as median percentiles of degraded hydrogels analyzed from 12 different images. Scale bar 200 µm. *p value < 0.05 tested in Wilcoxon non-parametric test.

Based on the fact that PEG-Fb hydrogels can be easily degraded upon incubation with collagenase (Cohen et al., 2018), we speculated that such proteolytic environment in the lung could induce the degradation of microspheres that would be beneficial in the liberation of the cell for therapeutic purposes. To address this hypothesis, we designed an *ex vivo* experiment to examine if microspheres could be degraded during incubation with extracts of bacteria-infected lungs. The illustration of the designed experiment is presented in Figure 5A; Mice were infected intranasally with a lethal dose of Kim53 *Y. pestis* strain (100LD50). After 48 h, the inflammation in the lungs was well-established with increased egression of immune cells, leading to inflammation and tissue damage, whereas mock infected lungs had clear alveoli with defined air spaces (Figures 5B,C). Consequently, respiratory bacterial infection induced the secretion of metalloproteases, such as MMP3, 7, 8, 9, 14 and pro-inflammatory cytokines, such as TNFα and IL1β in the infected lungs (Figures 5D,E). At that time, the lungs were isolated, mashed and filtered to discard cells and bacteria, and the sterile extracts were introduced to encapsulated J774 macrophages. After 7 days in culture, we observed degradation of hydrogels and cell release that was significantly more prevalent (2-3 times greater) after incubation with lung extracts of infected mice compared to mock (Figures 5F–H). The released cells adhered to the culture surface and continued to grow and proliferate (Figure 5G, red arrows), demonstrating complete release and consolidation on the culture surface.

### Flowing encapsulated macrophages through human respiratory tract airways-on-chip models

**FIGURE 6:**
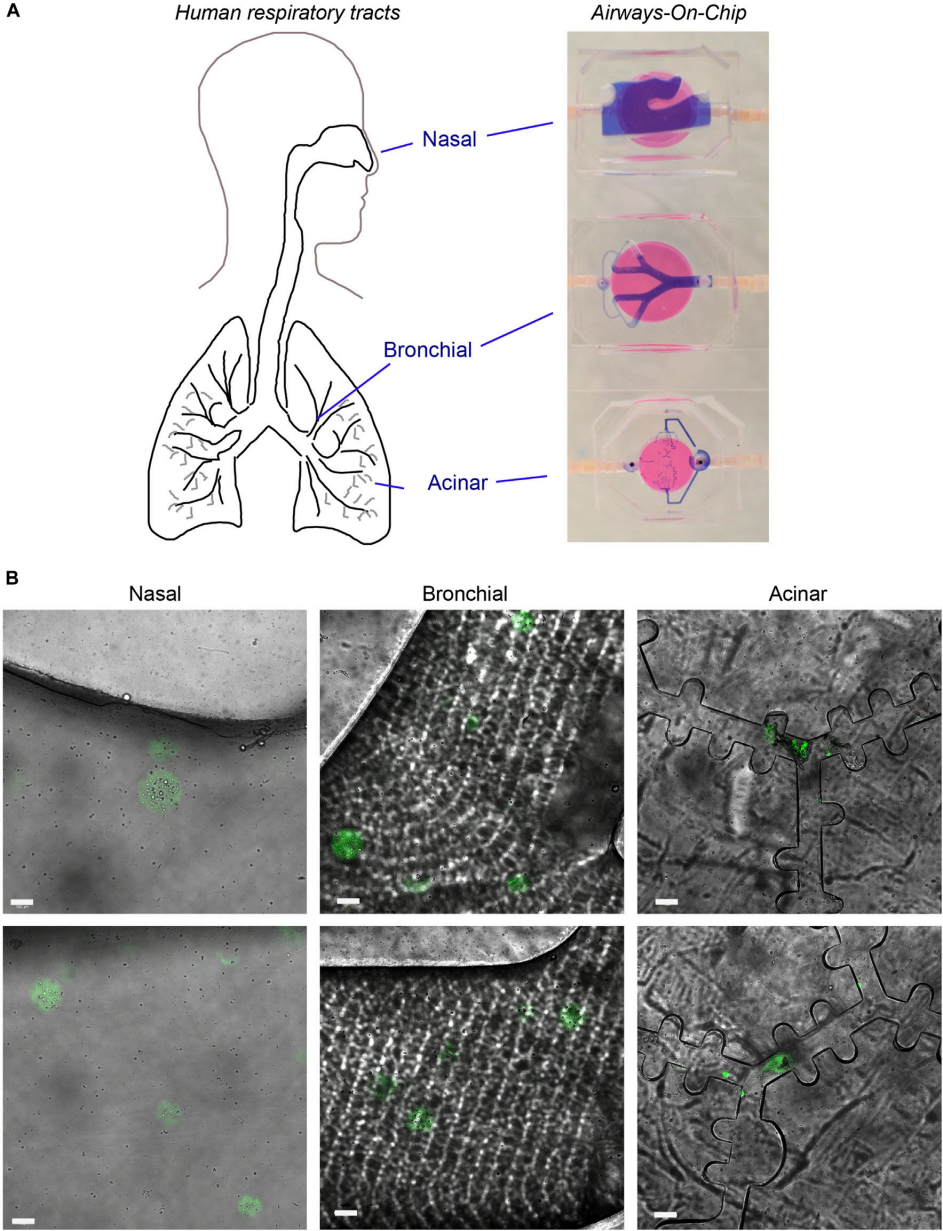
Microsphere delivery to human respiratory tract models. **(A)** An illustration of the human respiratory tracts: nasal, bronchial and alveolar and the parallel Airways-on-chip models. The models were injected with trypan blue to demonstrate the flow inside the compartments. **(B)** Confocal images of nasal, bronchial and alveolar compartments showing Calcein-stained microspheres (green). Two representative images from each compartment from three independent experiments are presented. Scale bar 100 µm.

To demonstrate the feasibility to deliver microspheres to respiratory tracts for therapeutic applications, we utilized a recently developed multi-compartment Airways-on-chip model of the human respiratory tracts (Nof et al., 2022). This model was designed to mimic anatomically the flows in respiratory zones and included three compartments with inlet channels: nasal (4 mm diameter), bronchial branches (between 2.2 and 1.25 mm diameter) and alveoli (100 µm height x 170 µm width). This multicompartment model has been previously used to demonstrate viral-laden airflow transmission through the respiratory system’s cellular landscape (Nof et al., 2022). Here we used this system to mimic intranasal administration of encapsulated cells in microspheres in fluid, to analyze microspheres delivery along the respiratory tracts. To that end, the multicompartment models were serially connected one to another with linking pipelines (illustration in Figure 6A), and encapsulated macrophages were streamed (200 microspheres/ml) in a constant flow rate (10 ml/min) using a hydraulic pump for 20 min. To mimic the physiologically airflow at the acinar model, a Y-joint tube-to-tube connector was added in the outlet of the bronchial part to reduce the flowrate fed. To allow easy monitoring in a confocal microscope, the macrophages were pre-stained with Calcein dye. Using both brightfield and fluorescent filters, we observed microspheres throughout all compartments: the nasal, bronchial as well as the alveolar (Figure 6B), demonstrating the feasibility to deliver microcarriers in a fluidic environment to human lungs.

## Discussion

With recent advances in the field of cell-therapy, there is a growing demand to improve delivery systems with the aim to increase engraftment efficiency. This study examined a novel immune cell delivery approach that is premised on cell encapsulation in hydrogel microspheres. We utilized a semi-synthetic PEG-Fb hydrogel scaffold that has been applied in different therapeutic studies, and exhibited high biocompatibility to various cell types, including preserving the viability and functionality of the cellular cargo. Using an emulsion-based photo-crosslinking reaction, we demonstrated co-encapsulation of two alveolar cell types (epithelial TC-1 cells and MH-S macrophages) that were equally dispersed inside the microspheres. The co-encapsulation of two different types of cells would have an added advantage when a close interaction between the cells is important to preserve cellular functionality (Belardi et al., 2020; Almet et al., 2021).

We proposed that the delivery of the microsphere carriers to inflamed and injured lung tissues would confer protection to the encapsulated cells and would be advantageous over administration of free cells without a carrier. Specifically, damaged lungs after an acute infection with infectious pathogens, such as *Yersinia pestis*, could obtain therapeutic benefit from exogenous macrophage delivery using the microspheres. The exposure of bacterial endotoxin, a “danger” signal to the innate immune response, induced a burst pro-inflammatory cytokine response in free non-encapsulated macrophages, whereas encapsulated macrophages were more protected and had only a mild response at intermediate endotoxin levels (10 ng/ml). The reduced activation of encapsulated macrophages may be explained by a possible restrictive effect of the microsphere structure on the branched molecular structure of LPS that allows only part of the LPS to penetrate the shell (Ghaly et al., 2011), whereas at high concentrations of 100 ng/ml more LPS can penetrate. Another explanation may be related to the composition of the hydrogel, made from natural fibrinogen, which mimics extracellular matrix and provides a physical support to the encapsulated cells (Coburn et al., 2011). It is most likely that the increase in TNFα secretion per cell (both encapsulated and non-encapsulated) after 96 h under 100 ng/ml LPS is related to the state of cell activation, rather than to cell density inside the microspheres. Interestingly we observed that the encapsulated cells became elongated and migrated outside the hydrogel microsphere upon exposure to LPS. It has been previously shown that LPS activate macrophage migration (Thorley et al., 2007), however, high levels of activation inhibited phagocytosis capabilities important for apoptotic cell clearance and resolution of inflammation (Feng et al., 2011). Therefore, the PEG-Fb hydrogels provided a protective encapsulating microenvironment for the macrophages, tempering their response to bacterial endotoxin stimulation as well as enhancing the migratory phenotype of the macrophage towards outgrowth from the microspheres.

Using an *ex vivo* animal model of *Yersinia pestis* airway infection in mice, 48-h post infection we observed a profound damage to the alveoli airways characterized with increased recruitment of immune cells and tissue damaged that was accompanied with pro-inflammatory cytokine and metalloprotease secretion. We postulated that such inflamed conditions would induce the degradation of the microsphere scaffolds and release the encapsulated cells. The incubation of microsphere *ex vivo* with filtered whole lung extract of infected mice enhanced the degradation of the microspheres and the release of the encapsulated cells, which subsequently adhered to the culture surface. Hence, in addition to the bacterial-induced mechanism of migration out of the microspheres, the proteolytic environment of infected whole lung extracts can further induce hydrogel degradation and promote the release of the encapsulated macrophages. Both mechanisms of microsphere degradation and enhanced migration of encapsulated cells could be beneficial towards the targeted delivery of therapeutic cells to infected lungs.

As a proof of concept, to demonstrate the feasibility of delivering microspheres to human respiratory tracts for therapeutic applications, we successfully flowed microspheres loaded with macrophages to Airways-on-chip models of human respiratory tracts including the nasal, bronchial and acinar airways. To mimic intra nasal administration, the microspheres were transported in a fluidic environment and were observed in all model compartments. This microsphere delivery demonstration was examined in clear airways. Different disease conditions such as lung congestion, may influence normal airways trafficking and consequently microsphere delivery that could be restricted to the first two compartments of the lung. Yet, in such conditions, encapsulated cell could be released following microsphere degradation and farther migrate to the acinar compartment.

In conclusion, this *ex vivo* study demonstrated the feasibility of using PEG-Fb hydrogel microspheres for topical immune cell delivery to respiratory tracts. We examined essential parameters including the conditions for encapsulation that preserve cell viability and functionality and physical flow rate to respiratory tracts. The interaction of encapsulated macrophages with bacterial environment was simulated *in vitro*, showing the added value of encapsulation over free cells that could be degraded and release the cells at site of inflammation. Taken together, this study paves the way for further *in vivo* investigations of immune cell-based delivery for tissue repair of pulmonary diseases.

## Data Availability

The raw data supporting the conclusion of this article will be made available by the authors, without undue reservation.

## References

[B1] Almany L. Seliktar D. (2005). Biosynthetic hydrogel scaffolds made from fibrinogen and polyethylene glycol for 3D cell cultures. Biomaterials 26, 2467–2477. 10.1016/j.biomaterials.2004.06.047 15585249

[B2] Almet A. A. Cang Z. Jin S. Nie Q. (2021). The landscape of cell–cell communication through single-cell transcriptomics. Curr. Opin. Syst. Biol. 26, 12–23. 10.1016/j.coisb.2021.03.007 33969247 PMC8104132

[B3] Artzy-Schnirman A. Zidan H. Elias-Kirma S. Ben-Porat L. Tenenbaum-Katan J. Carius P. (2019). Capturing the onset of bacterial pulmonary infection in acini-on-chips. Adv. Biosyst. 3, 1900026. 10.1002/adbi.201900026 PMC7611792 32648651

[B4] Belardi B. Son S. Felce J. H. Dustin M. L. Fletcher D. A. (2020). Cell–cell interfaces as specialized compartments directing cell function. Nat. Rev. Mol. Cell Biol. 21, 750–764. 10.1038/s41580-020-00298-7 33093672

[B5] Birman T. Seliktar D. (2021). Injectability of biosynthetic hydrogels: consideration for minimally invasive surgical procedures and 3D bioprinting. Adv. Funct. Mat. 31, 1–17. 10.1002/adfm.202100628

[B6] Coburn J. Gibson M. Bandalini P. A. Laird C. Mao H.-Q. Moroni L. (2011). Biomimetics of the extracellular matrix: an integrated three-dimensional fiber-hydrogel composite for cartilage tissue engineering. Smart Struct. Syst. 7, 213–222. 10.12989/sss.2011.7.3.213 22287978 PMC3266370

[B7] Cohen N. Toister E. Lati Y. Girshengorn M. Levin L. Silberstein L. (2018). Cell encapsulation utilizing PEG-fibrinogen hydrogel supports viability and enhances productivity under stress conditions. Cytotechnology 70, 1075–1083. 10.1007/s10616-018-0204-x 29468479 PMC6021291

[B8] Dikovsky D. Bianco-Peled H. Seliktar D. (2006). The effect of structural alterations of PEG-fibrinogen hydrogel scaffolds on 3-D cellular morphology and cellular migration. Biomaterials 27, 1496–1506. 10.1016/j.biomaterials.2005.09.038 16243393

[B9] Elias-Kirma S. Artzy-Schnirman A. Das P. Heller-Algazi M. Korin N. Sznitman J. (2020). *In situ*-like aerosol inhalation exposure for cytotoxicity assessment using airway-on-chips platforms. Front. Bioeng. Biotechnol. 8, 91. 10.3389/fbioe.2020.00091 32154228 PMC7044134

[B10] Feng X. Deng T. Zhang Y. Su S. Wei C. Han D. (2011). Lipopolysaccharide inhibits macrophage phagocytosis of apoptotic neutrophils by regulating the production of tumour necrosis factor α and growth arrest-specific gene 6. Immunology 132, 287–295. 10.1111/j.1365-2567.2010.03364.x 21039473 PMC3050451

[B11] Fuoco C. Cannata S. Bottinelli R. Seliktar D. Cossu G. Gargioli C. (2012a). Autologous progenitor cells in a hydrogel form a supernumerary and functional skeletal muscle *in vivo* . J. Tissue Eng. Regen. Med. 6, 116.

[B12] Fuoco C. Salvatori M. L. Biondo A. Shapira-Schweitzer K. Santoleri S. Antonini S. (2012b). Injectable polyethylene glycol-fibrinogen hydrogel adjuvant improves survival and differentiation of transplanted mesoangioblasts in acute and chronic skeletal-muscle degeneration. Skelet. muscle 2, 24. 10.1186/2044-5040-2-24 23181356 PMC3579757

[B13] Ghaly T. Rabadi M. M. Weber M. Rabadi S. M. Bank M. Grom J. M. (2011). Hydrogel-embedded endothelial progenitor cells evade LPS and mitigate endotoxemia. Am. J. Physiology-Renal Physiology 301, F802–F812. 10.1152/ajprenal.00124.2011 PMC3191807 21775481

[B14] Hussell T. Bell T. J. (2014). Alveolar macrophages: plasticity in a tissue-specific context. Nat. Rev. Immunol. 14, 81–93. 10.1038/nri3600 24445666

[B15] Levy Y. Flashner Y. Zauberman A. Tidhar A. Aftalion M. Lazar S. (2010). “Protection against plague afforded by treatment with polyclonal αLcrV and αF1 antibodies,” in The challenge of highly pathogenic microorganisms (Dordrecht: Springer), 269–274. 10.1007/978-90-481-9054-6_29

[B16] Li S.-H. Lai T. Y. Y. Sun Z. Han M. Moriyama E. Wilson B. (2009). Tracking cardiac engraftment and distribution of implanted bone marrow cells: comparing intra-aortic, intravenous, and intramyocardial delivery. J. Thorac. Cardiovasc. Surg. 137, 1225–1233.e1. 10.1016/j.jtcvs.2008.11.001 19379996

[B17] Lin K.-Y. Guarnieri F. G. Staveley-O’Carroll K. F. Levitsky H. I. August J. T. Pardoll D. M. (1996). Treatment of established tumors with a novel vaccine that enhances major histocompatibility class II presentation of tumor antigen. Cancer Res. 56, 21–26. 8548765

[B18] Mitrousis N. Fokina A. Shoichet M. S. (2018). Biomaterials for cell transplantation. Nat. Rev. Mat. 3, 441–456. 10.1038/s41578-018-0057-0

[B19] Nof E. Zidan H. Artzy-Schnirman A. Mouhadeb O. Beckerman M. Bhardwaj S. (2022). Human multi-compartment airways-on-chip platform for emulating respiratory airborne transmission: from nose to pulmonary acini. Front. Physiol. 13, 853317. 10.3389/fphys.2022.853317 35350687 PMC8957966

[B20] Peled E. Boss J. Bejar J. Zinman C. Seliktar D. (2007). A novel poly (ethylene glycol)–fibrinogen hydrogel for tibial segmental defect repair in a rat model. J. Biomed. Mat. Res. A 80, 874–884. 10.1002/jbm.a.30928 17072852

[B21] Rufaihah A. J. Johari N. A. Vaibavi S. R. Plotkin M. Kofidis T. Seliktar D. (2017). Dual delivery of VEGF and ANG-1 in ischemic hearts using an injectable hydrogel. Acta biomater. 48, 58–67. 10.1016/j.actbio.2016.10.013 27756647

[B22] Seeto W. J. Tian Y. Winter R. L. Caldwell F. J. Wooldridge A. A. Lipke E. A. (2017). Encapsulation of equine endothelial colony forming cells in highly uniform, injectable hydrogel microspheres for local cell delivery. Tissue Eng. Part C. Methods 23, 815–825. 10.1089/ten.tec.2017.0233 28762895

[B23] Sheikh A. Y. Huber B. C. Narsinh K. H. Spin J. M. van der Bogt K. de Almeida P. E. (2012). *In vivo* functional and transcriptional profiling of bone marrow stem cells after transplantation into ischemic myocardium. Arterioscler. Thromb. Vasc. Biol. 32, 92–102. 10.1161/ATVBAHA.111.238618 22034515 PMC3241895

[B24] Suzuki T. Arumugam P. Sakagami T. Lachmann N. Chalk C. Sallese A. (2014). Pulmonary macrophage transplantation therapy. Nature 514, 450–454. 10.1038/nature13807 25274301 PMC4236859

[B25] Sznitman J. (2021). Revisiting airflow and aerosol transport phenomena in the deep lungs with microfluidics. Chem. Rev. 122, 7182–7204. 10.1021/acs.chemrev.1c00621 34964615

[B26] Thorley A. J. Ford P. A. Giembycz M. A. Goldstraw P. Young A. Tetley T. D. (2007). Differential regulation of cytokine release and leukocyte migration by lipopolysaccharide-stimulated primary human lung alveolar type II epithelial cells and macrophages. J. Immunol. 178, 463–473. 10.4049/jimmunol.178.1.463 17182585

[B27] Tomashefski J. F. Jr (2000). Pulmonary pathology of acute respiratory distress syndrome. Clin. chest Med. 21, 435–466. 10.1016/s0272-5231(05)70158-1 11019719

[B28] Vagima Y. Gur D. Erez N. Achdout H. Aftalion M. Levy Y. (2020). Influenza virus infection augments susceptibility to respiratory *Yersinia pestis* exposure and impacts the efficacy of antiplague antibiotic treatments. Sci. Rep. 10, 19116. 10.1038/s41598-020-75840-w 33154422 PMC7645720

[B29] Vagima Y. Levy Y. Mamroud E. (2019). “Monitoring of neutrophil recruitment to mice lungs during pneumonic plague,” in Pathogenic Yersinia (New York, NY: Humana), 141–150. 10.1007/978-1-4939-9541-7_10 31177436

[B30] Vagima Y. Zauberman A. Levy Y. Gur D. Tidhar A. Aftalion M. (2015). Circumventing *Y. pestis* virulence by early recruitment of neutrophils to the lungs during pneumonic plague. PLoS Pathog. 11, e1004893. 10.1371/journal.ppat.1004893 25974210 PMC4431741

[B31] Wang X. Rivera-Bolanos N. Jiang B. Ameer G. A. (2019). Advanced functional biomaterials for stem cell delivery in regenerative engineering and medicine. Adv. Funct. Mat. 29, 1809009. 10.1002/adfm.201809009

